# Statistic Experience Based Adaptive One-Shot Detector (EAO) for Camera Sensing System

**DOI:** 10.3390/s18093041

**Published:** 2018-09-11

**Authors:** Xiaoning Zhu, Bojian Ding, Qingyue Meng, Lize Gu, Yixian Yang

**Affiliations:** 1 Information Security Center, Beijing University of Posts and Telecommunications, Beijing 100876, China; buptdbj@bupt.edu.cn (B.D.); mengqingyue@bupt.edu.cn (Q.M.); glzisc@bupt.edu.cn (L.G.); yxyang@bupt.edu.cn (Y.Y.); 2 Guizhou Provincial Key Laboratory of Public Big Data, Guizhou University, Guiyang 550025, China

**Keywords:** remote sensing, convolutional neural network, image recognition, object detection, prior boxes

## Abstract

Object detection in a camera sensing system has been addressed by researchers in the field of image processing. Highly-developed techniques provide researchers with great opportunities to recognize objects by applying different algorithms. This paper proposes an object recognition model, named Statistic Experience-based Adaptive One-shot Detector (EAO), based on convolutional neural network. The proposed model makes use of spectral clustering to make detection dataset, generates prior boxes for object bounding and assigns prior boxes based on multi-resolution. The model is constructed and trained for improving the detection precision and the processing speed. Experiments are conducted on classical images datasets while the results demonstrate the superiority of EAO in terms of effectiveness and efficiency. Working performance of the EAO is verified by comparing it to several state-of-the-art approaches, which makes it a promising method for the development of the camera sensing technique.

## 1. Introduction

Cameras are used to capture scalar phenomena in the form of images or videos, which provide more detailed and impressive data of the physical world [1]. In recent years, the camera sensing system has tended to be more automatic and intelligent. Compared with traditional cameras, a camera sensing system contains a communication interface, memory, an operating system and a processor. Aiming at facilitating the follow-up image inspection and judgement, the current development trend is to integrate the image sensor and detection algorithm into the same system in order for it to become modular as a camera sensor [2]. In most situations, imaging sensors are sensitive, accurate and well responding to objects. As a result, the detection of objects is important for the improvement of camera performance. This issue is most pronounced in the field of image processing and recognition. Due to the progress in machine learning methodologies, object detection models dramatically outperform manual detection, which exploit the best detection strategy by applying different algorithms. Neural network is one such principle with the potential to be beneficial for object detection optimizing.

The architecture of a convolutional neural network (CNN) was initially designed to take advantage of the 2D structure of an input signal [3,4]. Nevertheless, in line with the grand step in ImageNet Large Scale Visual Recognition Challenge (ILSVRC), the re-utilization of CNN is most pronounced due to its high precision in object detection and [5]. Thereafter, studies on CNN models were conducted and the recognition accuracy kept updating simultaneously [6,7,8,9]. Concretely, the mean average precision (mAP) and detection speed as the target detection parameters for open source dataset like PASCAL VOC and COCO keep improving continually. Researchers prefer to give the first priority to the advance in selective search (SS) [10] and region-based convolutional network (R-CNN) [11]. Fast R-CNN, which is a state-of-the-art detection algorithm, is capable of providing real-time processing via very deep convolutional networks regardless of the region proposal [12,13]. Further, Region Proposal Network (RPN) is proposed where a multimodal convolutional network is applied to jointly predict objectness and localization on a fusion of image and temporal motion cues [14]. Accordingly, for each Faster R-CNN, it is necessary to hold both a proposal network and a detection network, which is too sophisticated to optimize the model [15]. Specifically, You Only Look Once (YOLOv2) is a current well-employed method, with recent publications exploring the promise of using a hierarchical view of object classification and combining distinct datasets together. The basic idea of YOLO is to divide the image into smaller grid cells with each grid cell predicting bounding boxes and confidence for those boxes and class probabilities [16,17]. Unlike YOLO, the SSD architecture combines predictions from multiple feature maps at different resolutions in the network, which naturally handles objects of different sizes and improves detection quality [18].

The use of current-proposed detection model is, however, still limited, primarily because the single dataset, integrating difficulty, slow processing speed and low accuracy. For these reasons, the research is still ongoing to mitigate the deficiencies. In this research, we propose an object detection model EAO, statistic experience based adaptive one-shot detector, with the property of end-to-end detection. To enlarge the current detection dataset, a strategy for making a detection sample from a classification sample is proposed. Meanwhile, a detection dataset, namely ImageNet iLOC, for image processing models training and testing is constructed. The spectral clustering and ResNet methodology are integrated for image processing. The remaining part of this paper is organized as follows:

The background knowledge of spectral clustering, ResNet and stochastic gradient descent is depicted in Section 2. Section 3 describes the framework of EAO as well as the working principle in detail. Section 4 shows the results achieved in object detection experiments and the analysis of the model. The research findings and future planning for camera sensing system are presented in Section 5.

## 2. Preliminaries

### 2.1. Spectral Clustering

Clustering is one of the important techniques in the machine learning portfolio, particularly in unsupervised settings [19]. Spectral clustering is characterized with spectral graph theory—a field dedicated to graph studying via the eigenvalues and eigenvectors of matrices naturally associated with them [20]. In other words, spectral clustering splits data by means of graph partition using the weighted adjacency matrix and its spectrum [21,22]. Therefore, by exploiting the major components in the data revealed by the spectrum, spectral clustering results in a more powerful representation of data in feature space, so as to facilitate data clustering [23].

Assuming that we are given *n* samples, each datum $x_{1},x_{2},\ldots ,x_{n}$ is in a sample space $x_{i}\in X$. The symmetric weighted adjacency matrix $W\in R^{n\times n}$ encodes the pairwise similarity where $0\leq w_{ij}\leq 1$. Aiming at obtaining a symmetric normalized weight matrix, the normalized graph Laplacian is computed as follows.

(1) $$L=D^{-\frac{1}{2}}WD^{-\frac{1}{2}}$$

Seeing that $w_{ij}\in W$, we shall also define

(2) $$L_{ij}=\frac{w_{ij}}{{D_{ii}D_{jj}}^{-\frac{1}{2}}}$$

where *D* is a diagonal matrix and $D_{ii}=\sum_{j=1}^{n}w_{ij}$. Suppose that there are *n* orthonormal eigenvectors corresponding to the eigenvalues $\lambda_{1},\lambda_{2},\ldots ,\lambda_{n}\left(\lambda_{1}\geq \lambda_{2}\geq \ldots \geq \lambda_{n}\right)$ of *L*.

Considering the eigenvectors selection process, a new matrix $U\in R^{n\times k}$ is formed by stacking the first largest *k* eigenvectors from *L*. Thus, we have:

(3) $$L\left(k\right)=UU^{T}$$

Mathematically, the eigenvectors inherit information from the adjacency matrix as the new expression enhances the ample properties in the feature space. The samples lay in feature space are either clustered closer or repulsed further apart, which lead to a more prominent cluster in the feature space [24,25]. Thus, the data dimension is significantly reduced.

### 2.2. ResNet

To the best of our knowledge, features from deeper networks tend to obtain a better working performance than that extracted from shallow networks [26]. Whereas, the gradient fading with the network depth increasing comes to be the greatest technical challenge. In 2015, He et al. proposed the deep residual framework named ResNet [27]. Currently, the depth of the ResNet has already reached an astonishing level of 1001 layers and apparently, the depth can also be increased [28].

Since it is impossible to fit the output mapping with the stacked layer, He et al. focus on fitting the residual mapping and pushing the residual to zero. In [29], the idea of “shortcut connections” is first put forward for making the inputs identity mapping. Meanwhile, to improve the processing speed, He et al. carefully tune the layer sizes to balance the computation between various sub-networks [30].

### 2.3. Stochastic Gradient Descent

Stochastic gradient-based optimization is one of the important issues in mathematical resolution of scientific studies. Stochastic gradient descent (SGD), which is the core part of all the stochastic methods, is capable of processing large-scale problems in a way that clearly outperforms classical optimization methods [31,32]. Specifically, SGD proves its efficiency and reliability in recent advances in neural networks [33,34]. Generally, a convolutional network contains alternating layers of convolution and pooling (i.e., subsampling). Stochastic-pooling, which is one of the wide-spread pooling methods, shows the capability of the deep learning process to prevent the issue of over-fitting [35].

ADAM, proposed by D. P. Kingma and J. L. Ba is an algorithm for automatically tuning the learning rate in SGD [36]. Commonly, the loss function is defined for a single training example for SGD and is shown in Equation (4):

(4) $$L_{SGD}\left({\vec{x}}_{p}\right)=\frac{1}{2}{\left(y_{p}-y\left({\vec{x}}_{p}\right)\right)}^{2},p=1,2,\ldots ,P$$

The weight updating of the model is expressed in Equations (5)–(8).

(5) $$w^{-new}=w^{-old}-\alpha \cdot y_{i}\cdot \left(y_{p}-y\left({\vec{x}}_{p}\right)\right)$$

(6) $$c_{j}^{i_{new}}=c_{j}^{i_{old}}-\beta \cdot \frac{h_{j}^{i}}{\sum_{j=1}^{m_{i}}h_{j}^{i}}\cdot \left(y_{p}-y\left({\vec{x}}_{p}\right)\right)$$

(7) $$a_{j}^{i_{new}}=a_{j}^{i_{old}}-\gamma \cdot \frac{h_{j}^{i}}{\sum_{j=1}^{m_{i}}h_{j}^{i}}\cdot \left(c_{j}^{i}-y_{i}\right)\cdot \left(y_{p}-y\left({\vec{x}}_{p}\right)\right)\cdot \frac{2(x_{pi}-a_{j}^{i_{old}})}{b_{j}^{i}}$$

(8) $$b_{j}^{i_{new}}=b_{j}^{i_{old}}-\eta \cdot w_{i}\cdot \left(c_{j}^{i}-y_{i}\right)\cdot \frac{h_{j}^{i}}{\sum_{j=1}^{m_{i}}h_{j}^{i}}\cdot \left(y_{p}-y\left({\vec{x}}_{p}\right)\right)\cdot \frac{2(x_{pi}-a_{j}^{i_{old}})}{b_{j}^{i}}$$

where α, β,γ and η indicate learning rates of SGD, which influence the stability of the learning process. Nevertheless, based on the deployment of ADAM, the parameter update is simplified as:

(9) $$\theta^{new}=\theta^{old}-\kappa \cdot \frac{\hat{m}}{\sqrt{\hat{v}}+\varepsilon }$$

where κ is the learning rate, θ is the model parameter replacing the *a*, *b*, *c* and *w* and ∇ θ is the gradient from the loss function. ε is a hyper-parameter specifically for ADAM. Considering the computation efficiency, *m* and *v* are initialized as the 1st moment vector and the 2nd moment vector which are applied to be the exponential moving average of ∇θ and ${\left(\nabla \theta \right)}^{2}$ separately. The $\hat{m}$ and $\hat{v}$ designate the parameters after deviation correction. Computation of *m* and *v* are facilitated by using the following formula:

(10) $$\hat{m}=\frac{m}{1-\beta_{1}},\hat{v}=\frac{v}{1-\beta_{2}}$$

where $\beta_{1}$ and $\beta_{2}$ are exponential decay rates for the moment estimating, which are set during the model initialization.

## 3. EAO Object Detection Methodology

In this section, we describe EAO for image capture and processing, which can effectively address the difficulties in object detection tasks. In the proposed algorithm, all the samples are taken from the open access image dataset ImageNet, PASCAL VOC and COCO.

### 3.1. From Classification Dataset to Detection Dataset

Current object detection samples only occupy a small part of the datasets for other purposes (e.g., classification and tagging) [37,38]. Previous works have been focused primarily on obtaining an even higher accuracy of classification samples [6,39]. In such work, images are presented for machine learning model training and further testing [7,8]. Alternatively, object detection datasets are used as a secondary source due to their finite applications. For this reason, the object classification dataset holds great promise for effective learning of given targets after the labeling and recognizing process. Consequently, a strategy for converting a classification sample into a detection sample is devised. Consequently, we made the detection dataset, namely ImageNet iLOC, for network training and put forward the strategy for converting classification samples into detection samples.

Both ResNet and Spectral cluster provide good performances in the field of image classification and image segmentation. On the other hand, cropping [33,40] and warping [11,41] will inevitably bring deviation as well. According to the basic theory of deep learning and the experimental outcomes, the improvement in working performance and normalization definitely outweighs the working error. Spectral clustering is a kind of unsupervised learning method. In the case of image processing, spectral clustering is used to outline the object and further determine its relative position. In addition, the ResNet approach is employed for object classifying and matching.

Generally, an object set is defined as $X_{i}=\left\{c_{x},c_{y},w,h,category\right\}$ where $c_{x}$ and $c_{y}$ indicate the coordinate of the ground truth box center, the width *w* and height *h* characterize the size of ground truth box and category designate the object label. The ground truth box of the detection object is determined by spectral clustering. Specifically, we shall define the coordinate of left border *L* and bottom border *D* as the feature vectors for each sample. To start with, an input image sample is segmented into m×n pixels while the feature vectors can be expressed as $L=\{{\vec{l}}_{1},{\vec{l}}_{2},\ldots ,{\vec{l}}_{m}\}$ and $D=\{{\vec{d}}_{1},{\vec{d}}_{2},\ldots ,{\vec{d}}_{n}\}$. The spectral clustering algorithm is deployed on the aforementioned pixels to acquire each cluster, which represents one detection object $O_{j}$ and $O_{j}\in O=\left\{O_{1},O_{2},\ldots ,O_{M}\right\}$ where *O* stand for the object set in the image. In line with each cluster, the coordinate vector of each pixel point of the object is defined as $C^{j}=\left\{{\vec{c}}_{1}^{j},{\vec{c}}_{2}^{j},\ldots ,{\vec{c}}_{K}^{j}\right\}$. Therefore, each pixel point, with the maximum and minimum distance to the image boundary {L,D} can be calculated. Let ${\vec{t}}_{u}^{j}$, ${\vec{t}}_{d}^{j}$, ${\vec{t}}_{l}^{j}$ and ${\vec{t}}_{r}^{j}$ be the upper, bottom, left and right vertex of object contour respectively, we shall thus define

(11) $$T_{j}=\left\{{\vec{t}}_{u}^{j},{\vec{t}}_{d}^{j},{\vec{t}}_{l}^{j},{\vec{t}}_{r}^{j}\right\}$$

Moreover, the coordinates of the anchor box vertexes can be represented by $\left(x_{1},y_{1}\right)$, $\left(x_{2},\Delta y\right)$, $\left(\Delta x,y_{3}\right)$ and $\left(x_{4},y_{4}\right)$ which are furthest to the image boundary on each side.

(12) $${\vec{t}}_{u}^{j}{=}_{\kappa \in K}^{\max}distance\left(D,{\vec{c}}_{k}^{j}\right)\;{\vec{t}}_{d}^{j}{=}_{\kappa \in K}^{\min}distance\left(D,{\vec{c}}_{k}^{j}\right)\;{\vec{t}}_{l}^{j}{=}_{\kappa \in K}^{\max}distance\left(L,{\vec{c}}_{k}^{j}\right)\;{\vec{t}}_{r}^{j}{=}_{\kappa \in K}^{\min}distance\left(L,{\vec{c}}_{k}^{j}\right)$$

In Equation (12), the function distance() stands for calculating the distance of the vertex. Accordingly, the basis parameters *w*, *h* and $\left(c_{x},c_{y}\right)$ are obtained, i.e.,

(13) $$\begin{array}{c} w=x_{4}-\Delta x\\ h=y_{1}-\Delta y\\ (c_{x},x_{y})=\Delta x+w,\Delta y+h \end{array}$$

**Figure 1:**
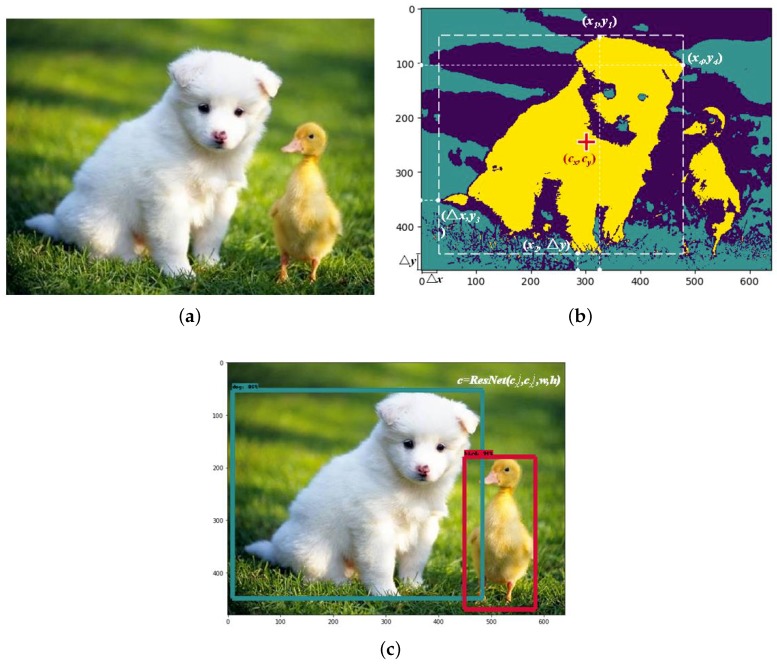
(**a**) Original image; (**b**) Anchor box defining; (**c**) Object recognition.

Hereafter, the image label is determined by the value of confidence via ResNet. In addition, following the results from Faster R-CNN, SSD, YOLO, etc., we conduct a considerable number of experiments on object detection. The confidence value is adjusted based on experimental outcomes. We find that 85% for the confidence threshold results in a best working capability. If and only if confidence≥0.85 the target ground truth outputs the label of $\left\{\left(c_{x}^{j},c_{y}^{j}\right),w,h,c\right\}$. Figure 1 presents an example of two target objects recognition for a particular image. The object recognition process with ImageNet iLOC is described as Algorithm 1.
**Algorithm 1** Generating object detection dataset based on spectral clustering**Input:** Classification sample *X* and the size of the dataset is *n*. **Output:** Detection object *D* 1:**function**ClassificationToDetection(*X*)2:    Initialization $k_{cluster}=6$3:    **for** i=1 to *n* **do**4:        Compute the left and bottom coordinates of sample $X_{i}$5:        Update the coordinate set {L,D}6:        Spectral clustering for detection object *O* based on sample $X_{i}$7:        **for** j=1 to $O_{M}$ **do**8:             Compute the upper vertex $t_{u}^{j}$, bottom vertex $t_{d}^{j}$, left vertex $t_{l}^{j}$ and right vertex $t_{r}^{j}$ of object $O_{j}$9:             Update the object coordinate $T_{j}=\left\{{\vec{t}}_{u}^{j},{\vec{t}}_{d}^{j},{\vec{t}}_{l}^{j},{\vec{t}}_{r}^{j}\right\}$ base on Equation (12)10:             Compute the width *w* and height *h* of the anchor box base on Equation (13)11:             Compute the center coordinate $\left(c_{x}^{j},c_{y}^{j}\right)$ of the anchor box base on Equation (13)12:             Update the positioning information of $O_{j}$ as $b_{j}=\left\{\left(c_{x}^{j},c_{y}^{j}\right),w,h\right\}$13:             Compute the parameter confidence of object $O_{j}$ as $c=ResNet(b_{j})$14:             **if** $\max_{c}\left(c_{i}\right)>0.85$ **then**15:                 Compute the ground-truth box of object $O_{j}$ as $gtb_{j}=\left\{\left(c_{x}^{j},c_{y}^{j}\right),w,h,c\right\}$16:                 Update the detection dataset with $gtb_{j}$17:           **else**18:                 continue19:           **end if**20:        **end for**21:    **end for**22:    **return** the new detection dataset *D*23:**end function**


### 3.2. Prior Box Generating

As a rule, objects are sought to be delineated before their identification [10]. In order to get a unique partitioning of the object, most bounding boxes are defined manually based on experience. In most cases, the bounding boxes lack statistical analysis, which restrain the intersection over union (IOU) overlapping [11,13]. Previous work has paid less attention to both the anchor box (Faster) and the default boxes (SSD). Box shape and number are taken from subjective estimation. For this reason, the ground-true box cannot be approached and the object cannot be detected. In this research, K-means++ is employed for box shape clustering. The objects can be better outlined. Further, the efficiency of bounding boxes is improved by revising the parameter *k*. Thereupon, we focus on the prior data from the novel datasets to accurately construct the bounding box. Providing the exploratory nature of the figure composition, the images contain different configurations regarding the color, shape, as well as texture of the objects. The goal of this stage is to take K-means++ clustering to detect the shape and size of the object prior box, and thus to improve the convergence speed and detection accuracy. Unlike K-means clustering employed in YOLO and DSSD, K-means++ gets an increasing number of cluster centers, which reduces the uncertainty of random selection and improves the clustering speed and accuracy [18,42,43].

**Figure 2:**
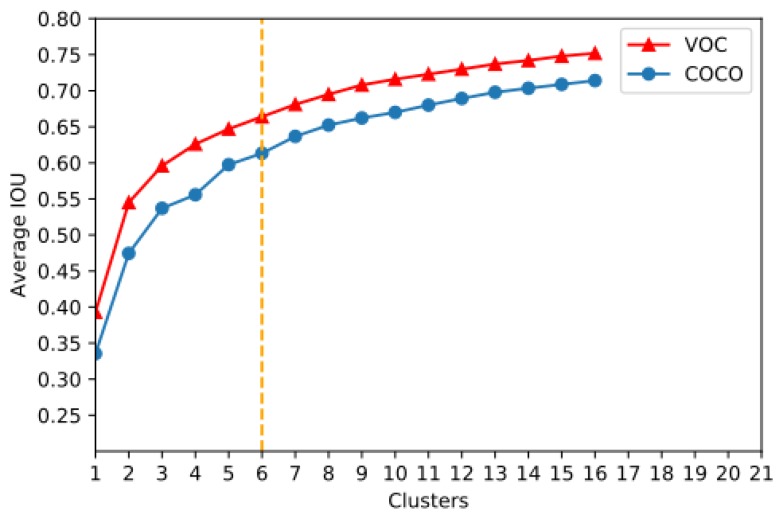
K-means++ clustering outcome for box number determination. IOU: intersection over union.

**Figure 3:**
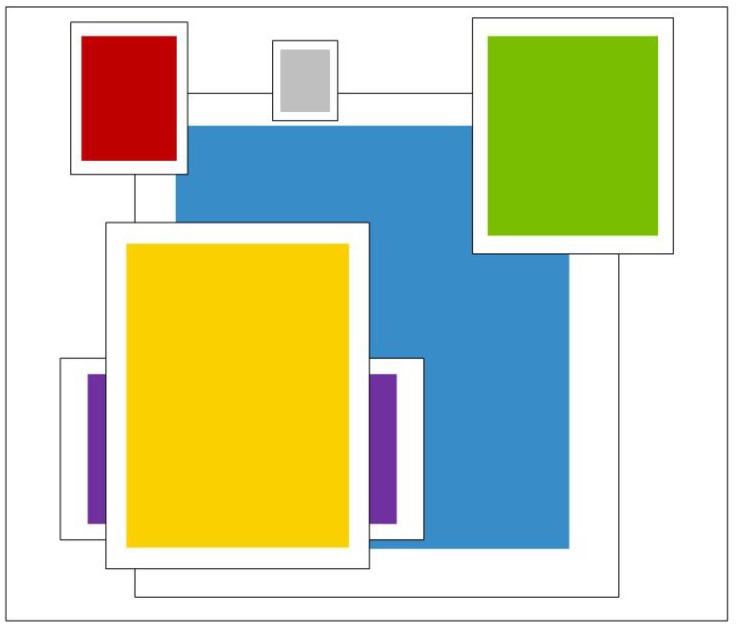
Clustering box dimensions on PASCLE VOC and COCO. We run k-means++ clustering on the dimensions of bounding boxes to get good priors for EAO model. Both sets of priors favor thinner, taller boxes while COCO has greater variation in size than PASCLE VOC.

Considering the model complexity and high recall, K=6 is adopted as a good tradeoff for further computing (Figure 2). Normally, the Euclidean distance is used to denote the distance between an element and the cluster center in the clustering algorithm. Note that the objects are of different sizes; the Euclidean distance cannot exactly reflect the object location in the image. We shall thus use a more appropriate form to demonstrate the distance, which is shown in Equation (14):

(14) d(box,centroid)=1−IOU(box,centroid)

where box represents the samples and centroid the cluster center. Function IOU outputs the overlapping ratio of this object to the cluster. Figure 3 shows the clustering result based on dataset ImageNet and COCO while most bounding boxes are of slender shapes. This involves boxing the object with the clustering outcome, which compares the same image via different bounding approaches. The bounding boxes from K-means++ clustering outcomes show better working performance, evidenced by a higher IOU overlapping proportion.

### 3.3. Multi-Resolution Feature Mapping

**Figure 4:**
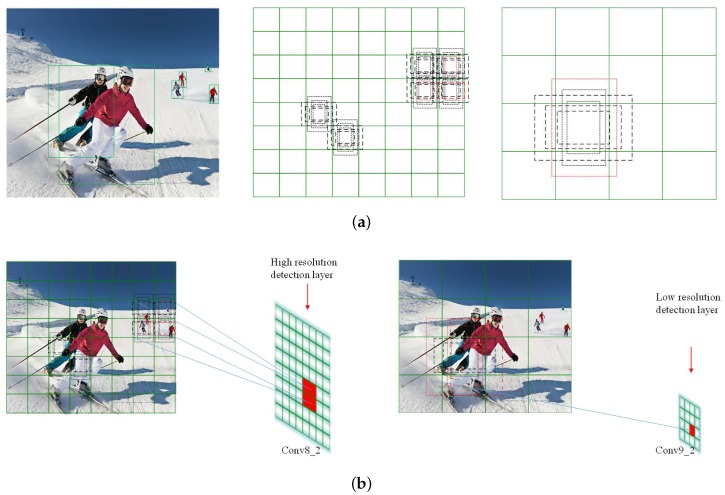
(**a**) Top prior boxes acquired from SSD; (**b**) Bottom prior boxes acquired from EAO.

Object capturing on real images is both laborious and time-consuming. The single-shot detector (SSD), which significantly outperforms other methods, combines the standard architecture and the auxiliary structure for high quality image classification. For each feature layer of size m×n, SSD assigns default boxes of different scales to every single cell. However, the bounding boxes cannot always accurately detect the object. The box in the red solid line is the specific ground truth. As depicted by an example in Figure 4a, for objects of different sizes, default boxes of high-resolution from SSD fail to cover the targets, which in turn increase the computing amount. Meanwhile, in the layers of low resolution, objects are missed because the boxes are too large. To best fit a specific layer, the resolution largely affects the number of boxes: for a high-resolution cell the default boxes are redundant while for a low-resolution one, more boxes need to be stacked and revise the prior boxes into slender shapes handled carefully. Note that the prior boxes are assigned due to the clustering results; the total number of boxes decrease with the overlapping ratio increases in Figure 4b.

In this research, we employ the feed-forward convolutional neural network to generate a fixed-size set of bounding boxes and a non-maximum suppression approach to determine the final detections, which optimizes the current SSD. According to Section 3.2, computation with K-means++ clustering for object detection is facilitated by taking K=6. For every single image, the number of target objects is so limited that the cost can be significantly reduced by assigning bounding boxes more precisely. Within one layer, each cell maps to a specific region of the original image, the size of which varies based on different resolution of the convolutional layer. Considering the image segmentation principle, bounding boxes can be defined due to layer resolution and the cell property. For this reason, we come up with the strategy that more bounding boxes should be assigned to the convolutional layer of lower resolution, and vice versa.

**Figure 5:**
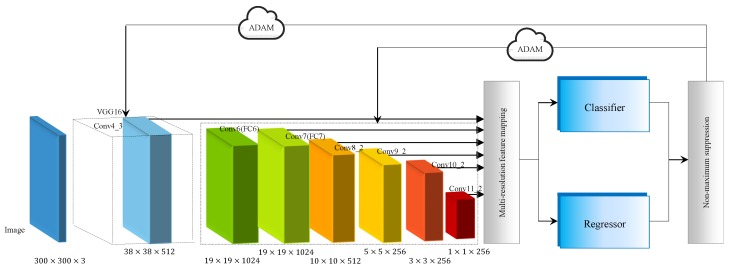
EAO architecture.

The model architecture is shown in Figure 5. EAO is initiated with standard VGG 16 as base network, which is a classical network for feature mapping [44]. Instead of using max-pooling, we employ the stochastic-pooling algorithm to address the issue of overfitting [45]. The multi-resolution detection layers are then integrated to seed the detection algorithm. The feature mapping is on the foundation of activation function Rectified Linear Unit (ReLU) to prevent gradient disappearance and results in faster learning [46]. By using the classical VGG 16 version, the basic layer conv4_3 is kept and the two fully connected layers, FC6 and FC7 are converted into typical 19×19 convolution layers [47]. The latter is utilized as the detection layer. As a result, the network is deployed by multiple feature maps, whose sizes are 38×38×512, 19×19×1024, 10×10×512, 5×5×256, 3×3 and 1×1×256. The convolutional layers decrease in size progressively. In each detection layer, a 3×3 convolution is applied to extract the feature of prior boxes. At each feature map cell, the offsets relative to the ground-truth box in the cell, as well as the conditional probabilities of the object category are picked. For a given cell with *k* prior boxes, we focus here on the four offsets involved with the ground-truth box. These layers are followed by a softmax classifier and a linear regressor, which predicts the category of the prior box and calculates the offset between the prior box and ground-truth box, respectively. Final detections are produced through non-maximum suppression step. Within the model, the random optimizer ADAM is taken for end-to-end training optimization.

Following the K-means++ clustering outcome, the shapes of bounding boxes for each layer are dedicatedly devised. The average IOU of a different detection model is presented (Table 1). With different bounding boxes assigned, a better average IOU of EAO is observed.

### 3.4. Training

The training process is originated from minimizing the loss of multitask objective function [48]. Supposing *x* is the convolution result for matching the predicted box *p* and the ground-truth box *g*, we introduce the classification loss (cls) and the regression loss (reg). Considering the multiple object categories, the overall objective loss function is a weighted sum of the classification loss and the regression loss:

(15) $$L\left(x,c,p,g\right)=\frac{1}{N}\left(L_{cls}\left(x,c\right)+\alpha L_{reg}\left(x,p,g\right)\right)$$

where *N* is the number of matched boxes, *c* is the multiple-class confidence and α is the weight term for controlling detection error. Typically, the regression loss is determined by $smooth_{L1}$ which characterizes the localization accuracy between the predicted box *p* and the ground truth [12].

One of the key processes gleaned from the Faster R-CNN is that the offset of the width (*w*), the height (*h*) and the central point ($c_{x},c_{y}$) can be obtained as the solution of regression. This idea is developed in our strategy for model evolution. We thus define the width, height and center of the predicted box, prior box and the ground-truth box. The offset vector between the prior box and the predicted box is ${\hat{t}}_{i}^{m}$ while that of the predicted box is *p* and the ground-truth box is *g*. Thereby, we have

(16) $$L_{reg}\left(x,p,g\right)=\sum_{i\in Pos}^{N}\sum_{m\in \left\{c_{x},c_{y},w,h\right\}}x_{ij}^{k}smooth_{L1}\left({\hat{t}}_{i}^{m}-{\hat{t}}_{j}^{m}\right)$$

in line with

$${\hat{t}}_{i}^{c_{x}}=\frac{p_{i}^{c_{x}}-d_{i}^{c_{x}}}{d_{i}^{w}},{\hat{t}}_{i}^{c_{y}}=\frac{p_{i}^{c_{y}}-d_{i}^{c_{y}}}{d_{i}^{h}}$$

(17) $${\hat{t}}_{i}^{w}=\log\frac{p_{i}^{w}}{d_{i}^{w}},{\hat{t}}_{i}^{h}=\log\frac{p_{i}^{h}}{d_{i}^{h}}$$

$$t_{j}^{c_{x}}=\frac{g_{j}^{c_{x}}-d_{i}^{c_{x}}}{d_{i}^{w}},t_{j}^{c_{y}}=\frac{g_{j}^{c_{y}}-d_{i}^{c_{y}}}{d_{i}^{h}}$$

$$t_{i}^{w}=\log\frac{g_{j}^{w}}{d_{i}^{w}},t_{j}^{h}=\log\frac{g_{j}^{h}}{d_{i}^{h}}$$

Note that the confidence loss is the softmax loss over multiple-class confidence *c*, the classification loss is defined as

(18) $$L_{cls}\left(x,c\right)=-\sum_{i\in Pos}^{N}x_{ij}^{p}\log\left({\hat{c}}_{i}^{p}\right)-\sum_{i\in Neg}\log\left({\hat{c}}_{i}^{0}\right)$$

together with

(19) $${\hat{c}}_{i}^{p}=\frac{\exp(c_{i}^{p})}{\sum_{p}\exp\left(c_{i}^{p}\right)}$$

After the prior boxes generated, each of the prior boxes with IOU overlap higher than 0.7 are selected as a positive proposal. On the other hand, negative training samples imply the overlap is lower than 0.3. The rest of the prior boxes are removed from inputs of the training model. Since most of the prior boxes are negative in practical use, the min-batch method is used to keep the ratio of matched to unmatched boxes to 1:1. For an image lacking positive samples, we employ the negative ones for supplementing. In this case, the ADAM algorithm is employed to improve the model accuracy based on offset revising.

## 4. Experiments

Experiments are conducted on three challenging datasets: PASCAL VOC 2007, PASCAL VOC 2012 and COCO to evaluate the working performance of EAO. All the state-of-the-art algorithms, i.e., SSD, YOLO and Faster R-CNN, are trained with image detection datasets. The dataset, namely Imagenet, is taken for model training. In this manuscript, we introduce the self-made dataset ImageNet iLOC for EAO training after training with traditional detection samples. The detection accuracy outperforms other methods. The performance evolution is presented in Section 4.4.2. All the testing datasets in this research are the same.

### 4.1. Dataset

PASCAL VOC2007: The PASCAL VOC project provides standardized labelled images for object recognition. Meanwhile, the evaluation of recognition method on these datasets can be achieved through its evaluation server. There are images of 20 classes in PASCAL VOC2007. In order to detect objects from a number of visual object classes in realistic scenes, we take 16,551 images from the VOC2007 training collection and validation collection for training and 4952 pieces of test data for testing.

PASCAL VOC2012: Compared to PASCAL VOC2007, the size of image dataset increased substantially. In PASCAL VOC2012, each training image is associated to an annotation file providing object class label for each object. In this stage, all the 10,991 images from the dataset are used to evaluate the capability of EAO.

MS COCO: MS COCO consists of over 10,000 image samples of 91 object classes which aims at gathering images of complex daily scenes containing common objects in their natural context. However, objects in COCO tend to be smaller than those in PASCAL VOC, detection boxes assigning can therefore be adjusted for different layers.

### 4.2. Evaluation Protocol

Mean average precision(mAP): For a given task, a classical evaluation protocol is to compute the precision/recall curve. Recall stands for the proportion of all positive examples ranked above a given rank while precision for the proportion of all examples above that rank from the positive class [49]. Formally, the mAP that indicates the shape of precision/recall outcome is proposed to evaluate the detection performance of PASCAL VOC. The mAP is specified by 11-points interpolated average precision, which is the average of the maximum precision for recall levels at a fixed set of uniformly-spaced recall values [0,0.1,0.2,…,1.0] [50]. The precision at each recall level *r* is interpolated by taking the maximum precision. The mAP is expressed as

(20) $$mAP=\frac{1}{11}\sum_{r\in \left\{0,0.1,\ldots ,1\right\}}P_{interp}\left(r\right)$$

together with

(21) $$P_{interp}(r)=\underset{\tilde{r}:\tilde{r}\geq r}{\max}p(\tilde{r})$$

where is p($\tilde{r}$) the measured precision at recall $\tilde{r}$.

IOU: IOU is taken as a standardized metric for characterizing the performance of an object detector on MS COCO. Considering that more small objects appear in images of MS COCO, we evaluate the model detection precision for IOU ∈[0.5:0.05:0.9] [11].

### 4.3. Implementation Details

We evaluate EAO in comparison to SSD, YOLO and Faster R-CNN. To start with, we fine-tune the model with ADAM optimizer and set $\beta_{1}=0.9,\beta_{2}=0.999,\varepsilon =1\times 10^{-8}$. The initial learning rate is 0.001. The exponential decay rates vary slightly for different datasets after the first-step iteration. Working parameters for distinguished datasets are given in Table 2.

For each dataset, the EAO with two different types of input, 300×300 and 512×512, is taken for training and testing. To further improve the recognition precision, we make and release the object classification dataset ImageNet iLOC with the proposed method in Section 3.1 and pre-train EAO with it. Subsequently, the model is applied to PASCAL VOC2012. The iterations for the pre-training process is fixed at 150k. However, this number differs in the second training step which are conducted on each dataset’s own training images. The differences depend on the size of dataset.

### 4.4. Results

#### 4.4.1. PASCAL VOC2007

The network is first evaluated on PASCAL VOC2007 dataset, which is a basic task for object detection. We use the initialization parameters for 40k iterations, then continue training for 20k iterations with revised parameters in Table 2. Table 3 summarises all the results comparing with some state-of-the-art approaches, in terms of mAP on all categories.

**Figure 6:**
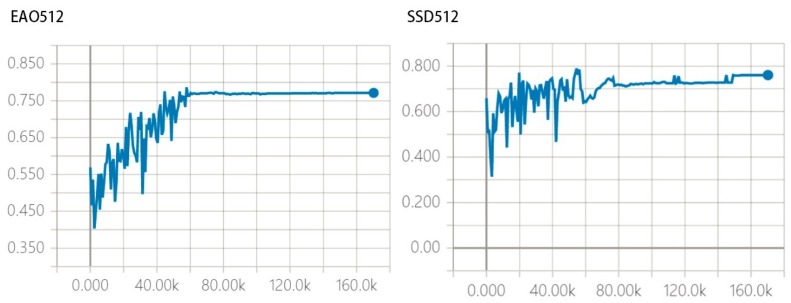
Working performance for EAO 512 and SSD512.

According to the testing outcome, our model trained with input 300×300, which keeps the same aspect ratio with SSD300, is of a higher accuracy (74.9% vs. 74.3% for SSD300). Our results denote EAO outperforms SSD when improving the original input size to 512×512. A 78.1%mAP is gained and the recall reaches 85–90%. In contrast with R-CNN, EAO is particularly effective due to the classifier and the regressor. To further assess the property of EAO from different resolution, we record the working process by employing analysis tool Tensorboard provided by Google tensorflow. With the multi-resolution network applied, a higher IOU can be obtained. Compared to SSD, EAO is highlighted with the convergence speed and the detection accuracy, which is expressed in Figure 6.

**Figure 7:**
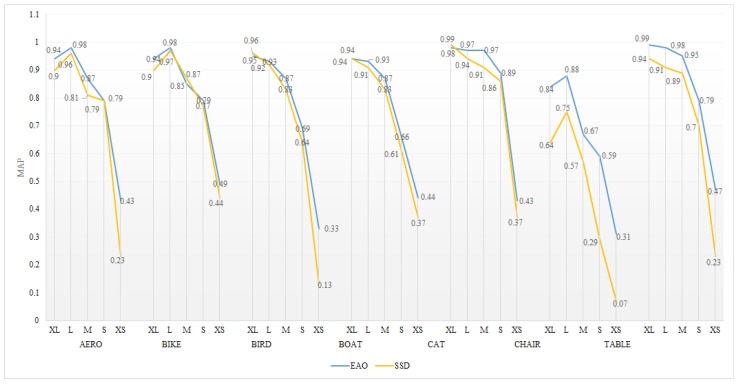
Working performance for EAO 512 and SSD512.

Most object recognition methods are limited in object detection since the object information is prone to be filtered through multi-layer convolution. On the one hand, Table 3 shows the detection precision for a small object is improved from a higher resolution network. On the other hand, prior boxes of various size are applied to different layers based on the pre-processing step in Section 3.1. Consequently, EAO provides higher robustness and detection precision than SSD (Figure 7). From prior boxes proposed to a high precision model, the oscillation processes stop after 60K and 80K times iteration for EAO512 and SSD512 separately. Crucially, EAO is amenable to efficiently stabilize the detection outcomes.

#### 4.4.2. PASCAL VOC2012

Aiming at obtaining a comprehensive evaluation of the proposed model, we assign to each model the training set provided by PASCAL VOC2012. Distinctively, we enlarge the training set of EAO, which contains both ImageNet iLOC data sets (produced in Section 3.1) and PASCAL VOC2012 trainval sets. Then all models are evaluated on PASCAL VOC2012 test set. The outcome similar to that from PASCAL VOC2007 is acquired (Table 4). According to the test results, the detection precision of EAO512 attains a 75.8 percent, which outperforms that of Faster R-CNN, YOLO and SSD via training from basic dataset. Moreover, by adopting the training sets from ImageNet, iLOC yields a 3.4 percent and a 3.6 percent improvement for EAO300 and EAO512, respectively. We also note that the performance of EAO300, with the new expansion data augmentation, is better than that of original EAO512 trained by PASCAL VOC2012. This happens because, given more exact detection objects, the model will be trained to work in a more accurate and faster mode. Incorporating the spectral clustering on objects and the bounding box prediction, our proposed object detection dataset is shown to greatly boost the detection accuracy. Likewise, it is noteworthy that the working performance on objects that are difficult to recognize is improved, like the boat and the bird.

#### 4.4.3. MS COCO

For the purpose of identifying our network on a more general, large-scale image dataset, we apply the models to MS COCO. Because objects in MS COCO tend to be smaller than those from PASCAL VOC, we train EAO with the initialization parameter for the first 140k iterations, followed by 40k iterations with the specifications in Table 2. Considering the bounding box generating strategy in Section 3.2, we use smaller prior boxes for all layers. The numbers and the sizes of the prior boxes for various layers are then determined based on the feature map principle.

**Figure 8:**
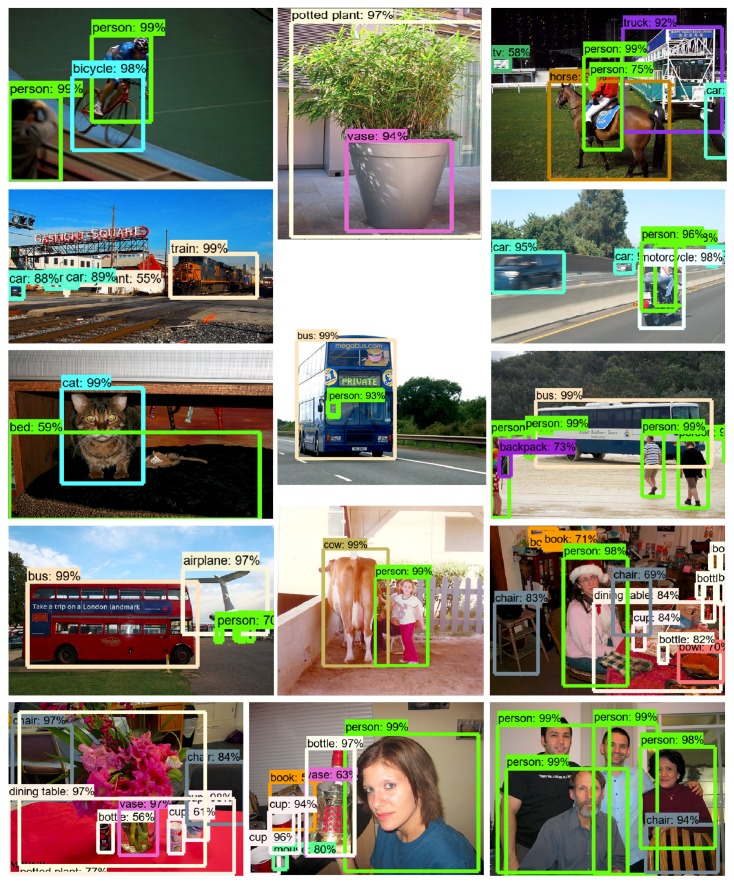
Some examples of EAO recognition results.

**Figure 9:**
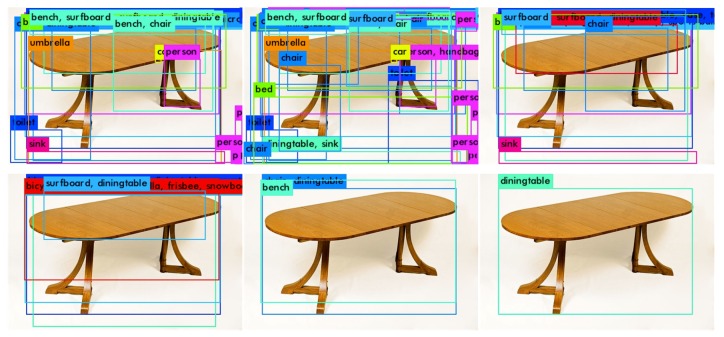
Convergence process of object recognition.

We take the whole trainval set for model training and the whole test set for testing. The results can be visualized in Table 5. The working performance of EAO300 is close to that of SSD512 while EAO512 exceeds the state-of-the-art methods on MS COCO dataset in all criteria. Specifically, both EAO300 and EAO512 significantly outperform other methods on mAP@0.5. The reason for this is most objects in MS COCO are relatively small while SSD and Faster R-CNN tend to fails to capture the delicate boundaries of small objects. The recognition results and the convergence process are presented in Figure 8 and Figure 9, respectively.

#### 4.4.4. Inference Time

As long as a large number of prior boxes are generated from our method, it is advisable to perform non-maximum suppression during inference. We filter most boxes with a confidence threshold of 0.05. The top 150 detections of each image are maintained by integrated non-maximum suppression and jaccard overlap of 0.45 per class. Note that 20 object classes belong to the PASCAL VOC dataset; the best result on each image is 1.3 ms with a 300×300 input while the total runtime on all detection layers is 1.9 ms. A quantitative comparison with other competing approaches is depicted in Table 6.

As long as a large number of prior boxes are generated from our method, it is advisable to perform non-maximum suppression during inference. We filter most boxes with a confidence threshold of 0.05. The top 150 detections of each image are maintained by integrated non-maximum suppression and jaccard overlap of 0.45 per class. Note that 20 object classes belong to PASCAL VOC dataset; the best result on each image is 1.3 ms. On 300×300 images, it takes all the six detection layers 1.9 ms on non-maximum suppression when running on a Xeon-E7-8800 v3 CPU with 2 GTX1080 Ti GPU and a Dell R740 server. A qualitative comparison with other competing approaches is depicted in Table 6. For the same image dataset, the Fast YOLO shows a decent inference time of 155 FPS but a poor detection precision. With the additional feature mapping layer applied to the region proposal network, the Fast R-CNN is slowed down by feature downsampling. Besides, outcomes of EAO are slightly better than SSD on both running time and detection precision. In particular, EAO300 achieves a mAP of 76.6 while maintaining a real-time speed of 61 FPS.

## 5. Discussion and Conclusions

This paper introduces EAO as an end-to-end convolutional network for object recognition, which is efficient and reliable to detect objects with a high precision for camera sensing. Aiming at facilitating the image processing, we make the detection dataset, namely ImageNet iLOC, via the spectral clustering method. This paper also proposes a detailed study of the prior box generating principle. Comprehensively, a multi-resolution network model for object detection is constructed and trained. Experiments are carried out on classical image datasets. By using the pretraining dataset ImageNet iLOC, an improved working performance is obtained. All experimental results are carefully analyzed. Compared to some state-of-the-art methods, the results validate the effectiveness of EAO and demonstrate the high efficiency in both the runtime and recognition accuracy.

Future work will address more complex situations where objects are presented in the camera-video form. The current model can be extended to a recurrent neural network by integrating it with other algorithms.

## Figures and Tables

**Table 1:** Network configuration parameters.

Box Generation	1	2	3	4	5	6	Sum of Boxes	Average IOU
Anchor box 256	-	-	-	-	-	256×9	2306	33.7%
Anchor box 512	-	-	-	-	-	512×9	4608	36.4%
Default box	38×38×6	19×19×6	10×10×6	5×5×6	3×3×6	1×1×6	11,640	54.7%
YOLO prior box	-	-	-	-	-	14×14×9	1764	66.5%
EAO SSE box †	38×38×3	19×19×3	10×10×6	5×5×6	3×3×9	1×1×9	5955	67.4%
EAO IoU box ‡	38×38×3	19×19×3	10×10×6	5×5×6	3×3×9	1×1×9	5955	**69.3%**

† The standard Euclidean distance as a distance calculation method for K-means++. ‡ The intersection over union (IoU) as a distance calculation method for K-means++.

**Table 2:** Working parameters for different datasets.

Working Parameters	$\beta_{1}$	$\beta_{2}$	ε	Learning Rate
PASCAL VOC 2007	0.75	0.777	$1\times 10^{-}8$	0.0007
PASCAL VOC 2012	0.9	0.999	$1\times 10^{-}8$	0.008
MS COCO	0.9	0.999	$1\times 10^{-}8$	0.0009

**Table 3:** PASCAL VOC2007 test detection results.

Method	mAP(%)	Aero	Bike	Bird	Boat	Bottle	Bus	Car	Cat	Chair	Cow	Table	Dog	Horse	Mbike	Person	Plant	Sheep	Sofa	Train	TV
Faster R-CNN	73.2	76.2	79	70.9	65.5	52.1	83.1	84.7	86.4	52.0	81.9	65.7	84.8	84.6	77.5	76.7	38.8	73.6	73.9	83.0	72.6
YOLO v2	73.4	86.3	82	74.8	59.2	51.8	79.8	76.5	90.6	52.1	78.2	58.5	89.3	82.5	83.4	81.3	49.1	77.2	62.4	83.8	68.7
SSD300	74.3	75.5	80.2	72.3	66.3	47.6	83.0	84.2	86.1	54.7	78.3	73.9	84.5	85.3	82.6	76.2	48.6	73.9	76.0	83.4	74.0
SSD512	76.8	82.4	84.7	78.4	73.8	53.2	86.2	87.5	86.0	57.8	83.1	70.2	84.9	85.2	83.9	79.7	50.3	77.9	73.9	82.5	75.3
EAO300	74.9	75.7	80.1	74.3	66.6	53.6	82.0	83.6	85.7	58.6	78.2	75.9	83.7	83.3	82.7	77.2	49.9	73.9	75.3	82.6	74.6
EAO512	**78.1**	85.7	**85.4**	**78.8**	71.3	**55.4**	84.9	87.3	86.9	**59.2**	82.8	74.3	85.9	**87.1**	**85.7**	**81.9**	**54.5**	**78.7**	74.1	**84.9**	**76.3**

**Table 4:** PASCAL VOC2012 test detection results.

Method	Data	mAP%	Aero	Bike	Bird	Boat	Bottle	Bus	Car	Cat	Chair	Cow	Table	Dog	Horse	Mbike	Person	Plant	Sheep	Sofa	Train	TV
Faster R-CNN	VOC07+12	70.4	84.9	79.8	74.3	53.9	49.8	77.5	75.9	88.5	45.6	77.1	55.3	86.9	81.7	80.9	79.6	40.1	72.6	60.9	81.2	61.5
YOLO v2	VOC07+12	57.9	77	67.2	57.7	38.3	22.7	68.3	55.9	81.4	36.2	60.8	48.5	77.2	72.3	71.3	63.5	28.9	52.2	54.8	73.9	50.8
SSD300	VOC07+12	72.4	85.6	80.1	70.5	57.6	46.2	79.4	76.1	89.2	53.0	77.0	60.8	87.0	83.1	82.3	79.4	45.9	75.9	69.5	81.9	67.5
SSD512	VOC07+12	74.9	87.4	82.3	75.8	59	52.6	81.7	81.5	90.0	55.4	79.1	59.8	88.4	84.3	84.7	83.3	50.2	78.0	66.3	86.3	72.0
EAO300	VOC07+12	73.6	86.8	83.5	74	58.6	51.8	82.0	83.6	85.7	53.4	75.9	58.2	82.1	83.3	82.7	77.9	48.9	77.4	68.4	82.4	68.2
EAO512	VOC07+12	75.8	76.5	84.3	75.4	66.5	53.6	83.8	87.2	85.6	58.3	78.3	77.9	83.5	85.9	82.4	78.3	55.3	77.5	70.2	80.9	74.3
EAO300	VOC07+12+iLOC	76.6	87.5	85.4	77.6	64.7	54.6	84.7	88.2	86.9	58.4	79.4	71.5	84.8	84.6	82.5	79.7	53.8	77.6	73.4	81.1	75.4
EAO512	VOC07+12+iLOC	**79.4**	**88.7**	**86.4**	**79.8**	**71.9**	**57.4**	**84.9**	**87.4**	88.9	**59.7**	84.8	77.3	87.9	**87.7**	84.1	**86.9**	**56.5**	**79.7**	**74.5**	85.9	**77.3**

**Table 5:** MS COCO test detection results.

Modes	Boxes	mAP@[0.5:0.95]	mAP@0.5	mAP@0.75
Faster R-CNN	RPN 300	21.6	41.5	20.9
ION	-	21.4	42.1	19.7
YOLO	96	20.4	43.5	19.4
SSD512	8732	23.5	43.9	23.7
EAO300	5955	21.7	44.1	23.6
EAO512	5955	**26.9**	**47.6**	**28.2**

**Table 6:** Testing results on inference time comparison.

Method	mAP	FPS	Number of Boxes
Faster R-CNN	73.2	7	300
YOLO	63.4	45	98
Fast YOLO	52.7	155	98
SSD300	72.1	58	7308
SSD512	75.1	21	200,097
EAO300	76.6	**61**	5955
EAO512	**79.4**	24	160,785
